# Capecitabine and irinotecan with bevacizumab 2-weekly for metastatic colorectal cancer: the phase II AVAXIRI study

**DOI:** 10.1186/s12885-015-1293-y

**Published:** 2015-04-29

**Authors:** Pilar Garcia-Alfonso, Manuel Chaves, Andrés Muñoz, Antonieta Salud, Maria García-Gonzalez, Cristina Grávalos, Bartomeu Massuti, Encarna González-Flores, Bernardo Queralt, Amelia López-Ladrón, Ferran Losa, Maria Jose Gómez, Amparo Oltra, Enrique Aranda, on behalf of the Spanish Cooperative Group for the Treatment of Digestive Tumors (TTD)

**Affiliations:** 1 Servicio de Oncología, Hospital Universitario Gregorio Marañón, C/Maiquez 7, 2nd floor, 28007 Madrid, Spain; 2 Servicio de Oncología, Hospital Virgen del Rocío, 41004 Sevilla, Spain; 3 Servicio de Oncología, Hospital Lleida Arnau de Vilanova, 25198 Barcelona, Spain; 4 Servicio de Oncología, Hospital Universitario de Burgos, 09005 Burgos, Spain; 5 Servicio de Oncología, Hospital 12 Octubre, 28041 Madrid, Spain; 6 Servicio de Oncología, Hospital General Universitario, 03011 Alicante, Spain; 7 Servicio de Oncología, Hospital Virgen de las Nieves, 18014 Granada, Spain; 8 Servicio de Oncología, ICO. Hospital. Josep Trueta, 17007 Gerona, Spain; 9 Servicio de Oncología, Hospital Nuestra Señora de Valme, 41014 Sevilla, Spain; 10 Servicio de Oncología, Hospital General de L’Hospitalet, 08906 Barcelona, Spain; 11 Servicio de Oncología, Hospital Puerta del Mar, 11009 Cádiz, Spain; 12 Servicio de Oncología, Hospital Virgen de los Lirios, 03804 Alicante, Spain; 13 Reina Sofía Hospital, University of Córdoba, Maimonides Institute of Biomedical Research (IMIBIC). Spanish Cancer Network (RTICC), Instituto de Salud Carlos III, Córdoba, Spain

**Keywords:** Irinotecan, Capecitabine, Bevacizumab, Metastatic colorectal cancer, Chemotherapy

## Abstract

**Background:**

The optimal sequence of chemotherapeutic agents is not firmly established for the treatment of metastatic colorectal cancer (mCRC). This phase II multi-centre study investigated the efficacy and tolerability of a standard capecitabine plus irinotecan (XELIRI) regimen with bevacizumab in previously untreated patients with mCRC.

**Methods:**

Patients received intravenous irinotecan 175 mg/m^2^ on day 1 and oral capecitabine 1000 mg/m^2^ (800 mg/m^2^ for patients >65 years of age) twice daily on days 2–8, followed by a 1-week rest, and bevacizumab 5 mg/kg as an intravenous infusion on day 1 every 2 weeks.

**Results:**

Seventy-seven patients were included in the intention-to-treat and safety populations. Progression-free survival at 9 months was 61%. The overall response and disease control rates were 51% and 84%, respectively. Median progression-free and overall survival times were 11.9 and 24.8 months, respectively. 48 patients (62%) had at least one grade 3/4 adverse event, the most common being asthenia, diarrhoea and neutropenia. Quality of life varied little over the study period with mean visual analogue scale general health scores ranging from 71 to 76 over cycles 1–11.

**Conclusion:**

Our study found irinotecan and capecitabine administered fortnightly with bevacizumab in patients with mCRC to be an effective and tolerable regimen.

**Trial registration:**

clinicaltrials.gov identifier NCT00875771. Trial registration date: 04/02/2009.

**Electronic supplementary material:**

The online version of this article (doi:10.1186/s12885-015-1293-y) contains supplementary material, which is available to authorized users.

## Background

According to the World Health Organisation’s most current statistics there are just over 12.6 million cases of cancer diagnosed each year. Colorectal cancer is the 5^th^ most common and accounts for 9.7% of all cancers [1]. Standard treatments for patients with metastatic colorectal cancer (mCRC) usually consist of combination chemotherapy based on fluorouracil or capecitabine plus either oxaliplatin or irinotecan, and a targeted agent such as bevacizumab, cetuximab or panitumumab [2,3]. The most commonly used chemotherapy regimens are fluorouracil with folinic acid plus oxaliplatin (FOLFOX), fluorouracil with folinic acid plus irinotecan (FOLFIRI), capecitabine plus oxaliplatin (XELOX), and capecitabine plus irinotecan (XELIRI). However, the optimal sequence of chemotherapeutic agents is not firmly established, and most patients will receive a fluoropyrimidine, irinotecan and oxaliplatin over the course of their treatment.

Randomised phase III studies have shown that the addition of bevacizumab to first- or second-line chemotherapy regimens extends overall survival and/or progression-free survival in patients with mCRC compared with chemotherapy alone [4-7]. Consequently, bevacizumab is indicated for the first- and second-line treatment of patients with mCRC [2,3].

Some uncertainty surrounds the most effective and tolerable schedule for administering irinotecan-based regimens such as XELIRI. The BICC-C and EORTC 40015 studies suggested that 3-weekly administration of irinotecan plus capecitabine can be associated with unacceptable gastrointestinal side effects [8,9], although both studies were confounded by the concomitant use of celecoxib, which is known to be associated with gastrointestinal toxicity. Consequently, different drug doses and administration regimens have been investigated with the aim of improving the tolerability of the combination of irinotecan and capecitabine [10-12].

We have previously shown that 2-weekly irinotecan plus capecitabine (irinotecan on day 1 every 2 weeks; plus capecitabine on days 1–7, followed by a week of rest) was effective and well tolerated in patients with mCRC [13]. Preclinical studies had shown this 2-weekly schedule, which is similar to the FOLFIRI schedule, to be more effective than the standard 3-weekly regimen and to allow the administration of higher capecitabine doses [14]. In our study, the adverse-event profile of XELIRI was acceptable, with asthenia, nausea, vomiting and diarrhoea being the most commonly observed grade 3/4 adverse events (occurring in 7–9% of patients). Dose delays and reductions occurred in <12% of patients for irinotecan and <5% of patients for capecitabine [13]. We subsequently incorporated bevacizumab into this regimen and demonstrated that this combination of a targeted agent with chemotherapy was effective and well tolerated in patients with mCRC [15].

The present phase II multicentre study was undertaken on behalf of the Spanish Cooperative Group for the Treatment of Digestive Tumors (TTD) to assess the efficacy and tolerability of 2-weekly regimen of irinotecan in combination with capecitabine plus bevacizumab in a larger population of previously untreated patients with mCRC.

## Methods

### Patients and study design

Patients ≥18 years of age with histologically proven, measurable mCRC that was not initially totally resected were included in this phase II open-label study. To be included, patients had an Eastern Cooperative Oncology Group (ECOG) performance status of 0–2 and could have had prior surgical treatment of their disease. No prior chemotherapy was allowed, other than adjuvant or neoadjuvant therapy completed at least 6 months before inclusion in the study; patients who received adjuvant therapy must not have progressed during or within 6 months of completing treatment. Additionally each patient was discussed by a multi-disciplinary team within each cancer centre to confirm their suitability for inclusion in the study.

Patients were not eligible for inclusion in the study if they had a history of central nervous system disease, psychiatric disability, clinically significant cardiac disease, lack of integrity of the upper gastrointestinal tract, malabsorption syndrome or inability to take oral medication. Exclusion criteria included any surgical procedures in the 28 days before the start of the study or if any surgery was scheduled to take place during the study. The use of oral anticoagulants or full-dose parenteral thrombolytic agents was not permitted, although low-dose warfarin was allowed. Chronic treatment with high-dose aspirin or antiplatelet agents was not permitted.

Patients with creatinine clearance <30 mL/minute or serum creatinine >1.5 times the upper limit of normal (ULN) were excluded from the study, as were those with: an absolute neutrophil count <1.5 × 10^9^/L; platelet count <100 × 10^9^/L; haemoglobin <9 g/dL; International Normalised Ratio >1.5; total bilirubin >1.5 × ULN; alanine aminotransferase and/or aspartate aminotransferase >2.5 x ULN (or >5 × ULN in case of liver metastases); or alkaline phosphatase >2.5 × ULN (or >5 × ULN in case of liver metastases or >10 × ULN in case of bone metastases).

The study protocol (Study TTD-08-03; EudraCT: 2008-004688-20; clinicaltrials.gov identifier NCT00875771) was approved by the Spanish Medicine Agency as well as the Institutional Review Board and Ethics Committee of each participating site (for details please refer to the Additional file 1). Reference Ethic Committee: “Comité Ético de Investigación Clínica” of the Hospital Universitario de Burgos, Avda. del Cid, 96,09005 Burgos on January 2009. Study procedures were carried out in accordance with the Declaration of Helsinki and its subsequent amendments, and Good Clinical Practice guidelines. Written, informed consent was obtained from all patients before enrolment.

### Treatment

Treatment consisted of irinotecan 175 mg/m^2^ as an intravenous infusion on day 1 every 2 weeks, capecitabine 1000 mg/m^2^ (800 mg/m^2^ for patients >65 years of age) twice daily on days 2–8, followed by a 1-week rest, and bevacizumab 5 mg/kg as an intravenous infusion on day 1 every 2 weeks. Treatment was continued until disease progression, unacceptable toxicity or patient withdrawal.

The doses of the chemotherapeutic agents were modified appropriately in each cycle according to the occurrence of toxicities. Once a dose was reduced, doses were not increased in subsequent cycles. If two dose reductions were sanctioned as a result of toxicity, patients experiencing the same complications were withdrawn from the study unless they had achieved an objective response to treatment, in which case the decision to continue treatment was left to the judgment of the investigator. If chemotherapy was delayed, administration of bevacizumab was also delayed. If the administration of chemotherapy was delayed for more than 2 cycles, the patient was withdrawn from the study. If irinotecan was discontinued, capecitabine and bevacizumab were to be continued unless unacceptable toxicity was observed. Similarly, if either capecitabine or bevacizumab were interrupted, treatment with the remaining agents could be continued at the investigator’s discretion.

### Assessments

The response to treatment was assessed using the radiological RECIST criteria [16] at 6-cycle intervals until the disease progressed or the patient died. No independent radiological review committee was established.

Adverse events were assessed at study visits and reported by patients. Adverse events were classified according to the National Cancer Institute Common Toxicity Criteria (CTC) version 3.0 [17]). All adverse events, regardless of their relation to the study treatment, were followed until resolution even if patients had withdrawn from the study.

Quality of life was measured using the EuroQoL 5-Dimensions (3-level) questionnaire (EQ-5D-3 L), a generic instrument used for measuring health status [18]. Quality of life assessments were performed at baseline, before each odd-numbered cycle (3, 5, 7, etc.) and in the 30 days following discontinuation of study therapy. A minimum of three assessments was required for the patient’s data to be included in the quality of life analysis. The EQ-5D-3 L assesses five different aspects of health (mobility, personal care, daily activities, pain/discomfort and anxiety/depression), each with three response categories. In addition, self-assessed general health was recorded using a 20 cm visual analogue scale (VAS) ranging from 0 (“worst imaginable health state”) to 100 (“best imaginable health state”).

### Statistical analyses

The primary endpoint of this phase II study was progression-free survival at 9 months. The secondary endpoints were: progression-free survival, overall survival, response rate, safety, resection rate and quality of life. Efficacy analyses were performed on the intention-to-treat population i.e. patients who received at least one dose of study medication. Safety analyses were performed on patients who received at least one dose of study medication (the safety population).

The sample size was based on a single-stage Fleming design, with p0 = 12% at 2 years (equivalent to a median progression-free survival of 8 months), p1 = 25% (equivalent to a median progression-free survival of 12 months), and an alpha error of 0.05 and a beta error of 0.01, resulting in a requirement for seventy-one evaluable patients. Allowing for a 10% dropout rate, seventy-nine patients were planned to be recruited.

Survival analyses were performed using Kaplan–Meier methodology; 95% confidence intervals (CIs) were calculated for the primary and secondary outcomes. Qualitative variables were described using absolute and relative frequencies; quantitative variables were described with means, medians and standard deviation (SD). All analyses were performed using SPSS version 17.0 (SPSS Inc. Released 2008. SPSS Statistics for Windows, Version 17.0. Chicago: SPSS Inc.).

## Results

### Patients

A total of eighty-one patients were enrolled in the study at twelve Spanish centres between 14 April 2009 and 20 April 2010. Four patients failed the screening process, with three violating entry criteria; one patient was hospitalised prior to initiation of treatment and could not be treated with the study regimen. The remaining seventy-seven patients received treatment and were included in the intention-to-treat and safety populations.

Patient characteristics are summarised in Table 1. A total of sixty-five patients had relevant comorbidities, the most common being hypertension in 32 patients (42%, twenty-seven of whom were taking antihypertensive agents) and 14 patients (18%) with diabetes mellitus. Twenty-one (27%) patients were ≥70 years of age. Fifty patients (65%) had undergone surgical resection of the primary tumour. Of the remaining twenty-seven patients who did not have surgical resection, four presented with intestinal perforation or occlusion.

**Table 1:** **Patient characteristics at baseline (N =77)**

**Sex**	***N***	**%**
Male	51	66.2
Female	26	33.8
Median age, years (range)	65 (41–81)	
**ECOG performance status**	***N***	**%**
0	46	59.7
1	28	36.4
2	3	3.9
**Location of primary tumour**	***N***	**%**
Rectum	24	31.2
Colon	41	53.2
Colon and rectum	12	15.6
**No. of lesions**	***N***	**%**
1	6	7.8
2	13	16.9
≥3	58	75.4
**Prior therapy**	***N***	**%**
Surgery	50	64.9
Adjuvant chemotherapy	27	35.1
Radiotherapy	9	11.7
**Metastatic site**	***N***	**%**
Liver	48	62.3
Lung	42	54.5
Local	18	23.4
Other	27	35.1
**Organs with metastases**	***N***	**%**
1	31	40.3
2	32	41.6
≥3	14	18.2

*Abbreviation: ECOG* Eastern Cooperative Oncology Group.

Tumour KRAS status was determined in seventy-one patients (92%); thirty-six patients (51%) had wild-type KRAS tumours and thirty-five (49%) had mutant KRAS tumours.

### Treatment

Patients underwent treatment for a median of 6.2 months (range 0.4–21.6 months) and received a total of 1009 cycles (876 cycles of bevacizumab, 973 cycles of irinotecan and 982 cycles of capecitabine). A median of 12.0 cycles (range 1.0–43.0 cycles) was administered; the median number of cycles of irinotecan, capecitabine and bevacizumab administered were 12.0 (range 1.0–43.0), 12.0 (range 1.0–43.0) and 11.0 (range 1.0–33.0), respectively.

The median relative dose intensities were: bevacizumab 89%, irinotecan 85%, and capecitabine 89%. Absolute median dose intensities were: bevacizumab 2.1 mg/kg/week, irinotecan 77.5 mg/m^2^/week, capecitabine 1439 mg/day.

Treatment was delayed in fifty-seven patients (74%) resulting in delays in 160 of the 1009 cycles (16%). The most common reasons for delayed doses were: neutropenia (25 cycles; 16%), administrative reasons (23 cycles; 14%); diarrhoea (19 cycles; 12%) and patient decision (15 cycles; 9%).

Bevacizumab was delayed in 119 cycles (14%) in twenty-four patients (31%), most commonly as a result of thromboembolism (67 cycles; 56%), fistula (9 cycles; 8%), wound-healing complications (8 cycles; 7%), and surgery (9 cycles; 8%). The irinotecan dose was reduced or delayed in 78 cycles (8%) in thirty-seven patients (48%) most commonly as a result of asthenia (12 cycles; 15%), diarrhoea (13 cycles; 17%) and at the discretion of the investigator (22 cycles; 28%). The capecitabine dose was reduced or delayed in 85 cycles (9%) in forty-six patients (60%) primarily as a result of diarrhoea (18 cycles; 21%).

Most patients received more than one line of treatment with sixty-two patients (81%) receiving second-line therapy, twenty-eight of whom had second-line bevacizumab-containing regimens. Thirty-seven patients received third-line and later lines of therapy.

### Efficacy

**Figure 1:**
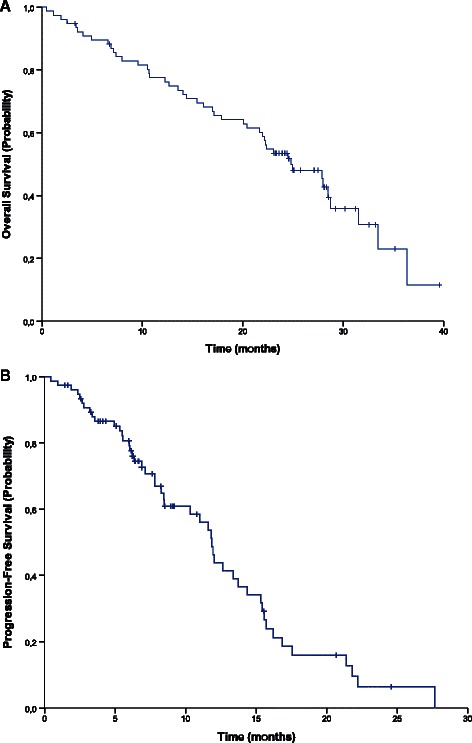
Overall survival **(A)** and progression-free survival **(B)** in patients treated with bevacizumab, capecitabine and irinotecan every 2 weeks.

Patients were followed for a median of 23.3 months (range 0.4–39.6 months). Efficacy outcomes are shown in Table 2 and Figure 1. Progression-free survival at 9 months (the primary endpoint) was 61% (95% CI: 48–73%). The median progression-free survival was 11.9 months (95% CI: 10.8–13.1 months) and median overall survival was 24.8 months (95% CI: 19.9–29.7 months). The overall response rate was 51% (95% CI: 39–62%) and the disease control rate was 84% (95% CI: 74–91%).

**Table 2:** **Efficacy (** ***N*** **= 77)**

Outcome	Months	95% CI
Median time to progression	11.9	10.8–13.1
Median progression-free survival	11.8	10.7–13.0
Median overall survival	24.8	19.9–29.7
**Response to treatment**	***N***	**%**
Complete response	4	5.2
Partial response	35	45.5
Stable disease	26	33.8
Progressive disease	5	6.5
Not evaluable	7	9.1
**Response rates**	**%**	**95% CI**
Overall response rate	50.6	39.1–62.0
Disease control rate	84.4	74.0–91.3

*Abbreviation: CI* confidence interval.

**Figure 2:**
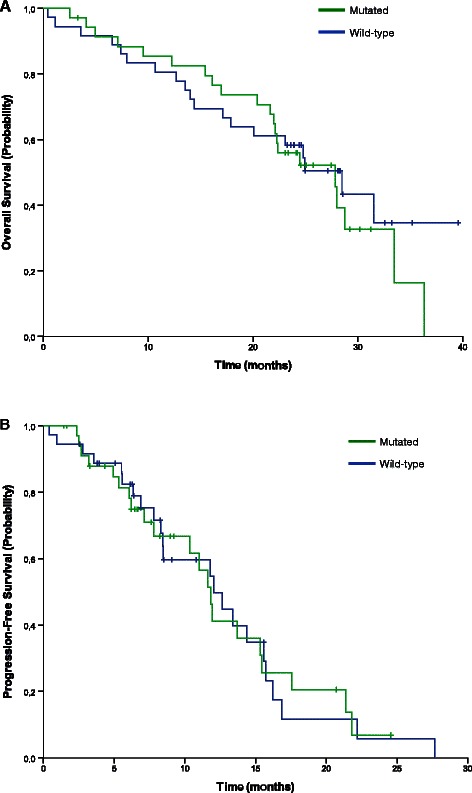
Overall survival **(A)** and progression-free survival **(B)** according to tumour KRAS status in patients treated with bevacizumab, capecitabine and irinotecan every 2 weeks.

Median progression-free survival was 12.0 months (95% CI: 6.6–17.5 months) in patients with wild-type KRAS tumours and 11.8 months (95% CI: 10.7–13.0 months) in those with mutant KRAS tumours (*P*= 0.985) (Figure 2). Overall survival was also similar in patients with wild-type and mutant KRAS tumours: 28.5 months (95% CI: 21.4–35.6 months) versus 27.9 months (95% CI: 21.4–34.3 months), respectively (*P*= 0.659; Figure 2). Confirmed response rates were 44.4% in patients with wild-type KRAS tumours and 37.1% in those with mutant KRAS tumours (*P* >0.05).

Seventeen patients (22%) had surgical resection of metastases during the study (65% liver metastases, 18% lung metastases, 12% peritoneal metastases and other sites). The median time to surgery after treatment initiation was 6.7 months. Twelve patients (71%) underwent R0 resection, three (18%) had an R1 resection and two (12%) were not evaluable. Thirteen of the seventeen patients who underwent surgical resection had further treatment (chemotherapy or immunotherapy). With respect to second-line chemotherapy six patients received post-surgical treatment with bevacizumab plus capecitabine/irinotecan and three patients received other bevacizumab-containing regimens. The remaining patients received a variety of other regimens that included oxaliplatin, cetuximab and panitumumab.

### Safety

To date, forty-five patients (58%) have died and thirty-two (42%) are still alive. The causes of death were: progressive disease *(N =* 35), adverse events *(N =* 7) and unknown *(N =* 3). Adverse events leading to death included: multi-organ failure *(N =* 2), gastrointestinal perforation *(N =* 2), respiratory and cardiac insufficiency due to chronic obstructive pulmonary disease *(N =* 1), myocardial infarction *(N =* 1) and respiratory insufficiency *(N =* 1). One of the gastrointestinal perforation events was considered to be related to treatment with bevacizumab; the two multi-organ failures were considered following discussion and scrutiny by the investigators to be related to capecitabine/irinotecan, rather than to disease progression as these events were reported in the context of toxicities.

A total of seventy-six patients (99%) had at least one adverse event related to treatment; forty-eight patients (62%) had at least one grade 3 or 4 adverse event. The most common grade 3 and 4 related adverse events were asthenia, diarrhoea and neutropenia (Table 3). Adverse events of special interest for bevacizumab are summarised in Table 4. Pulmonary embolism occurred in 10 patients, four of whom were >70 years of age; eight of these events were asymptomatic and two were symptomatic.

**Table 3:** **Grade 3 and 4 adverse events related to treatment occurring in >2% of patients (N = 77)**

Adverse event	Grade 3		Grade 4	
	*N*	%	*N*	%
Neutropenia	5	6.5	3	3.9
Febrile neutropenia	3	3.9	2	2.6
Alopecia	3	3.9	0	0
Hand–foot syndrome	4	5.2	0	0
Vomiting	4	5.2	0	0
Nausea	3	3.9	0	0
Diarrhoea	13	16.9	1	1.3
Asthenia	12	15.6	1	1.3

**Table 4:** **Grade 3 -5 adverse events of interest with bevacizumab (** ***N*** **= 77)**

Adverse event	Grade 3		Grade 4		Grade 5	
	*N*	%	*N*	%	*N*	%
Hypertension	2	2.6	0	0	0	0
Proteinuria	1	1.3	0	0	0	0
Gastrointestinal perforation	1	1.3	1	1.3	1	1.3
Arterial thrombosis	1	1.3	0	0	0	0
Deep vein thrombosis	1	1.3	1	1.3	0	0
Pulmonary thromboembolism	2	2.6	8	10.4	0	0

### Quality of life

EQ-5D-3 L questionnaires were completed by 70 patients (91%) at cycle 1, 58 of 70 patients (83%) at cycle 3, 49 of 64 patients (77%) at cycle 5, 38 of 55 patients (69%) at cycle 7, 36 of 47 patients (77%) at cycle 9, and 31 of 45 patients (69%) at cycle 11. After this point, the number of patients who completed questionnaires continued to decline, although some patients completed questionnaires until cycle 43.

**Figure 3:**
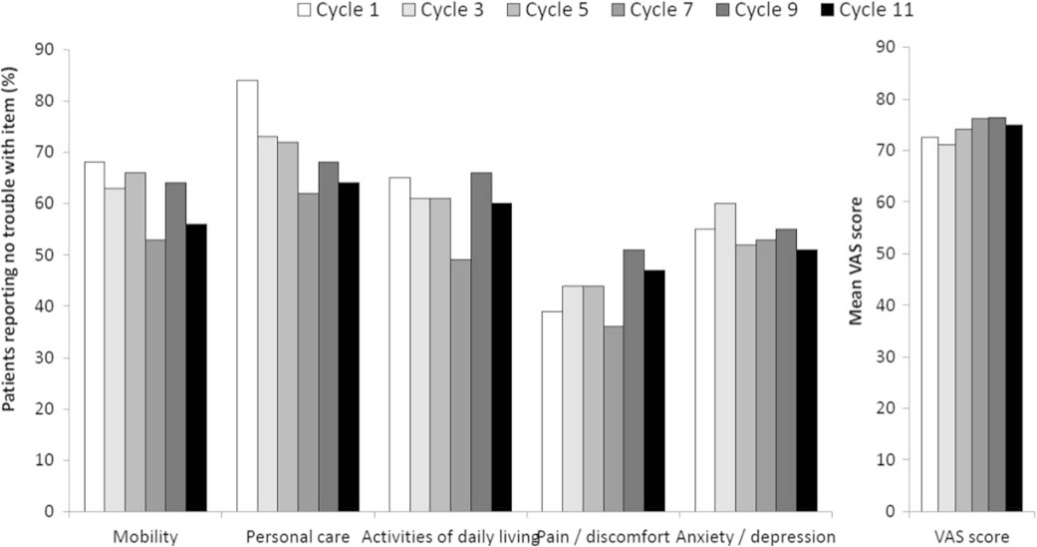
Changes in quality of life over the first 11 cycles of treatment as measured by the EuroQoL 5-Dimensions (3-level) questionnaire and patients’ VAS assessment of general health. Abbreviation: VAS = visual analogue scale.

Patient quality of life did not vary greatly over the study period (Figure 3). Most patients reported having no problems with mobility, patient care or activities of daily living during the first cycles of treatment. More than 50% of patients experienced pain or discomfort in the early cycles, although this proportion decreased as the study progressed. More than half of all patients reported feeling moderately or very anxious, or depressed throughout the study period. Mean VAS general health scores ranged from 71 to 76 over cycles 1–11 (Figure 3).

## Discussion

Considerable uncertainty surrounds the most effective use of irinotecan in combination with capecitabine for the treatment of patients with mCRC. Some studies have shown that irinotecan can be associated with significant gastrointestinal toxicities and, as a result, several doses and administration regimens have been investigated in order to maximise efficacy and tolerability.

This study has demonstrated that administering capecitabine–irinotecan plus bevacizumab every 2 weeks is a feasible and tolerable first-line treatment option for patients with mCRC. Cross-study comparisons, which should be made with caution, suggest that median progression-free survival and overall survival in the present study (11.8 months and 24.8 months, respectively) are similar to those reported in other phase II studies of bevacizumab plus XELIRI [19-22] and superior to those in which XELIRI was administered without bevacizumab [8,11,12]. This suggests that the efficacy of treatment was not compromised by the 2-weekly dosing schedule.

The safety profile of capecitabine–irinotecan plus bevacizumab administered every 2 weeks was comparable with reports from other phase II studies. We observed grade 3/4 diarrhoea in 18% of patients, which is similar to the 10–19% reported by others [19-22]; moreover, only one patient had grade 4 diarrhoea. Grade 3/4 neutropenia appeared to be somewhat less common than in other studies, occurring in 10% of patients in our study compared with 12–18% reported in those other studies.

Thromboembolic events have been reported as a complication of treatment with XELIRI–bevacizumab in the French FNCCLC ACCORD 13/0503 study, in which 24% of patients in the XELIRI–bevacizumab arm reported a venous thrombosis or pulmonary embolism [22]. In the present study, thromboembolic events were observed in 17% of patients, most of which were asymptomatic. Four of the eight grade 4 pulmonary emboli occurred in patients who were >70 years of age, amongst whom such events have been reported to be more common [23,24]. Indeed the incidence of thromboembolic events increased with age in bevacizumab-treated patients in the BRiTE registry, although the increase was not statistically significant after adjustment for baseline ECOG performance status, hypertension, the absence of anticoagulant therapy at baseline and prior history of thromboembolic events [25].

Median dose intensities were 89% for bevacizumab, 85% for irinotecan and 89% for capecitabine, suggesting that the regimen was generally well tolerated. These findings were within the confidence intervals of other studies that have reported on tolerability of the combination [21,22,26].

Response to treatment was not dependent on tumour KRAS status, as observed in other studies of bevacizumab plus chemotherapy in patients with mCRC [7,27-29]. As with the prognostic value of KRAS genotype study [29], we found that progression-free survival and overall survival were not extended significantly in KRAS wild-type genotypes over the mutant form. When interpreting these findings, it is important to note that the KRAS analysis was conducted retrospectively in a non-comparative trial.

Quality of life, as measured using the EQ-5D-3 L questionnaire, was maintained throughout the study, suggesting that treatment did not have a substantial negative impact on patients’ everyday activities. The evidence supports the validity of the EQ-5D-3 L tool in measuring quality of life in cancer patients [30], although the 5-level classification system, EQ-5D-5 L, has less ceiling effect and greater discriminative power [31] and we would consider using this tool in future studies. It is tricky to compare the VAS scores in Figure 3 with the EQ-5D-3 L scores. The subjective nature of the VAS scores gives some insight into the psychological tolerance of the effect of treatment on patients than the more objective EQ-5D-3 L. This self-perception of wellbeing improves over the course of the study. It would be interesting to investigate this (perhaps ‘placebo-like’) phenomenon more deeply in a separate study.

## Conclusions

In conclusion, this multicentre phase II study supports the use of irinotecan and capecitabine administered every 2 weeks with bevacizumab in patients with mCRC. The study included patients with multiple comorbidities, and elderly patients, and therefore indicates that this is an effective and tolerable regimen.

## Additional file

Additional file 1:
**Institutional Review Board and Ethics Committee.**

## Abbreviations

ECOG: Eastern Cooperative Oncology Group
FOLFIRI: Fluorouracil with folinic acid plus irinotecán
FOLFOX: Folinic acid plus oxaliplatino
mCRC: Metastatic colorectal cancer
TTD: Spanish cooperative group for the treatment of digestive tumors
XELIRI: Capecitabine plus irinotecán
XELIRI: Capecitabine plus irinotecán
XELOX: Capecitabine plus oxaliplatin
