# Supplementary material for: Capecitabine and irinotecan with bevacizumab 2-weekly for metastatic colorectal cancer: the phase II AVAXIRI study
Source: BMC Cancer. 2015 Apr 29;15:327. doi: 10.1186/s12885-015-1293-y (PMC4423590; doi:10.1186/s12885-015-1293-y)
Supplement: Additional file 1: — Institutional Review Board and Ethics Committee. [file 12885_2015_1293_MOESM1_ESM.docx]

**Institutional Review Board and Ethics Committee**

| **Participating sites** | **Institutional Review Board and Ethics Committee** |
| --- | --- |
| Hospital Universitario de Burgos | Hospital Universitario de Burgos. Comité Ético de Investigación Clínica  Reference Ethic Committee |
| Hospital Lleida Arnau de Vilanova (Lérida) | Hospital de Lleida Arnau de Vilanova. Comité Ético |
| Hospital General Universitario (Alicante) | Hospital General Universitario de Alicante. Comité Ético de Investigación Clínica |
| ICO. Hospital Josep Trueta (Gerona) | Hospital Dr. Josep Trueta. Comité Ético de Investigación Clínica |
| Hospital 12 de Octubre (Madrid) | Hospital 12 de Octubre. Comité Ético de Investigación Clínica |
| Hospital Virgen de las Nieves (Granada) | Hospital Virgen de las Nieves. Comité Ético de Investigación Clínica and Comité Autonómico de Ensayos Clínicos |
| Hospital Arnau de Vilanova (Valencia) | Hospital Arnau de Vilanova. Comité Ético |
| Hospital Puerta del Mar (Cádiz) | Hospital Universitario Puerta del Mar. Comité Ético de Investigación Clínica and Comité Autonómico de Ensayos Clínicos |
| Hospital Virgen del Rocío (Sevilla) | Hospital Virgen del Rocío. Comité Ético de Investigación Clínica and Comité Autonómico de Ensayos Clínicos |
| Hospital Nuestra Señora de Valme (Sevilla) | Hospital Nuestra Señora de Valme. Comité Ético de Investigación Clínica and Comité Autonómico de Ensayos Clínicos |
| Hospital General de L'Hospitalet (Barcelona) | Hospital General de L´Hospitalet. Comité Ético de Investigación Clínica |
| Hospital Universitario Gregorio Marañón (Madrid) | Hospital Universitario Gregorio Marañón. Comité Ético de Investigación Clínica |
| Hospital Virgen de los Lirios (Alicante) | Hospital Virgen de los Lirios. Comité Ético de Investigación Clínica |
